# MeMo: a hybrid SQL/XML approach to metabolomic data management for functional genomics

**DOI:** 10.1186/1471-2105-7-281

**Published:** 2006-06-05

**Authors:** Irena Spasić, Warwick B Dunn, Giles Velarde, Andy Tseng, Helen Jenkins, Nigel Hardy, Stephen G Oliver, Douglas B Kell

**Affiliations:** 1 School of Chemistry, Faraday Building, The University of Manchester, Manchester, M60 1QD, UK; 2 Department of Computer Science, The University of Wales, Aberystwyth, SY23 3DB, UK; 3 Faculty of Life Sciences, Michael Smith Building, The University of Manchester, Manchester, M13 9PT, UK

## Abstract

**Background:**

The genome sequencing projects have shown our limited knowledge regarding gene function, e.g. *S. cerevisiae *has 5–6,000 genes of which nearly 1,000 have an uncertain function. Their gross influence on the behaviour of the cell can be observed using large-scale metabolomic studies. The metabolomic data produced need to be structured and annotated in a machine-usable form to facilitate the exploration of the hidden links between the genes and their functions.

**Description:**

MeMo is a formal model for representing metabolomic data and the associated metadata. Two predominant platforms (SQL and XML) are used to encode the model. MeMo has been implemented as a relational database using a hybrid approach combining the advantages of the two technologies. It represents a practical solution for handling the sheer volume and complexity of the metabolomic data effectively and efficiently. The MeMo model and the associated software are available at .

**Conclusion:**

The maturity of relational database technology is used to support efficient data processing. The scalability and self-descriptiveness of XML are used to simplify the relational schema and facilitate the extensibility of the model necessitated by the creation of new experimental techniques. Special consideration is given to data integration issues as part of the systems biology agenda. MeMo has been physically integrated and cross-linked to related metabolomic and genomic databases. Semantic integration with other relevant databases has been supported through ontological annotation. Compatibility with other data formats is supported by automatic conversion.

## Background

The genome sequencing projects have shown our limited knowledge of gene function. For example, the sequencing of the well-studied model organism *Saccharomyces cerevisiae *has resulted in its total number of protein-encoding genes being estimated to be around 5,300 of which nearly 1,000 have a more or less unknown function [1]. Such gene functions can be studied with different omics technologies. While proteomics and transcriptomics have been widely used for this purpose, only recently have large-scale metabolomic experiments been applied [2,3]. These high-throughput technologies resulted in an exponential increase in the volume and the complexity of the omics data. Bearing in mind the limitations of a human brain, which can simultaneously handle only a dozen parameters [4], it becomes obvious that no significant progress in this type of biology can be made by relying on manual data analysis approaches alone.

The first step is to store such huge volumes of omics data in a machine-usable form. Data need to be formally structured and annotated so as to present biologists with hidden information in a way that can improve their existing knowledge [5]. Many biological DBs have been designed to address the needs of specific bio-communities (see the MBDC [6,7]). MBDC organises the given DBs hierarchically in order to simplify the process of finding a DB suitable for a given biological task. While other omics resources are listed at the top level of this hierarchy, metabolomic data sources are notably missing! In particular, no attempt has been made to store the growing flood of experimental data emerging from high-throughput metabolomic studies in some standardised format in a public DB [8].

In spite of the flood of omics data, we do not have an integrated understanding of cellular function, because existing research has mainly been focussed on individual levels (e.g. genomic, transcriptomic, proteomic, metabolomic, phenotypic) of the biological systems, neglecting the relations between them. SB has emerged as the appropriately holistic study of biological systems [9], based on a systematic integration of technology, biology and computation [10]. In order to achieve the simulation and functional prediction of complex biological systems and not just their isolated parts, it is essential to integrate various omics data resources describing different levels of cell organisation [11,12]. For example, EchoBASE [13] integrates data resulting from different types of omics (but not metabolomics) experiments and links them to information on specific *E. coli *genes. Obviously, in order to integrate all omics resources (including metabolomics), they need to be available individually in the first place.

The very first step is to develop and standardise all omics models and populate them with curated data. The transcriptomics community has made significant progress in this direction: the Microarray Gene Expression Data Society was founded in 1999 to facilitate the exchange of microarray data. These efforts resulted in a proposed standard for the MIAME [14] and the Microarray Gene Expression Markup Language [15]. Similarly, the Human Proteome Organisation founded the Protein Standards Initiative in 2002 [16] to coordinate the task of standardising the MIAPE [17] based on the PEDRo schema [18]. It is essential to take similar steps in metabolomics.

MIAMET has been suggested [19] in accordance with MIAME and MIAPE. MIAMET represents a natural language description or a checklist of the information necessary to provide context for metabolomic data. In 2003, the SMRS group was established with a similar agenda to work on a standard way of specifying the reports of metabolomic experiments [20]. As such, MIAMET and SMRS represent first positive steps towards a metabolomic data standard, but do not provide a complete formal description of the required information necessary for the development of supportive data handling systems. The Metabolomics Society founded in 2004 represents the most recent community-wide initiative to coordinate the efforts in standardising reporting structures of metabolomic experiments [21]. Five working groups have been founded to cover the key areas for describing metabolomic experiments: biological sample context, chemical analysis, data analysis, ontology and data exchange. In particular, the Metabolomics Society tries to build upon the existing project-driven attempts at modelling metabolomic experiments. For example, ArMet, originally proposed as a model for the description of metabolomic experiments specific for plants [22], is used to help define a more general standard.

In this paper, we suggest MeMo as a similar model for the formal representation of metabolomic data and the associated metadata (important for comparison, reproducibility and re-use of the results), initially for yeast but with a straightforward ability to extend it to other organisms. MeMo has largely re-used the high-level structure of ArMet. Some low-level components have been adapted or introduced to: (1) support different types of metadata needed for yeast studies, and (2) tackle practical issues of metabolomic data management. We implemented MeMo as an extended RDB to support the pressing need to store, manage and disseminate large amounts of curated metabolomic data efficiently. The long-term goal is to mine the content of the RDB using a variety of machine-learning techniques in order to extract hidden associations between genes and their functions implicitly represented by the metabolomic data [23,24].

## Construction and content

### Metabolomics model overview

The structure of the metabolomic data needs to be described by a suitable model, which can subsequently be translated into a DB schema. Entity-relationship diagrams [25] traditionally used to model RDBs have largely been overtaken by UML models [26], especially for bioinformatics applications. For example, UML has been used for the models described in [18,22,27-29]. UML is an OO modelling language which uses classes and relations as its main structuring mechanism. Classes are used to describe structural aspects of homogenous sets of objects by means of their attributes, operations and relations.

**Figure 1:**
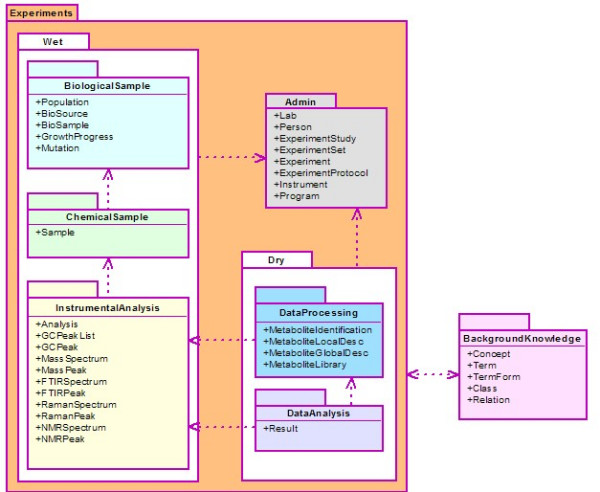
**Global overview of MeMo**. The global components of MeMo represented by UML packages.

MeMo describes the framework for metabolomic experiments in terms of the following seven components (Figure 1):

• **Admin. **Administrative and procedural data about an experimental framework (e.g. protocols, personnel, instruments).

• **Biological sample. **Metadata about the biological source examined (e.g. genotype, phenotype, biological sample).

• **Chemical sample. **Description of a sample prepared for instrumental analysis.

• **Instrumental analysis. **The results (e.g. raw data, mass spectrum) of analyzing a sample using an analytical instrument.

• **Data processing. **Identification of metabolites based on the results of instrumental analysis.

• **Data analysis. **Computational analysis of the analytical data and the associated metadata.

• **Background knowledge. **The background knowledge explicitly stored in the DB in a machine- readable form (e.g. gene classification, compound properties such as molecular weight, structure, reactions, etc.).

The experimental framework for metabolomics (described by the package called Wet experiments) can be divided into three components: biological, chemical and analytical. A biological sample is extracted from the biological source studied. It is used to produce a chemical sample to be employed in instrumental analysis.

To process the vast amounts of metabolomic data, additional analyses need to be performed *in silico *to extract knowledge. Therefore, a separate package is used to model Dry experiments, which covers the processing of raw analytical data (DataProcessing) and their computational analysis (DataAnalysis). Finally, the background knowledge package is used not only to cover all data coming from external sources covering different aspects of biological/chemical knowledge, but also the information inferred from the experimental data using different types of data analysis methods.

### Detailed metabolomics model

**Figure 2:**
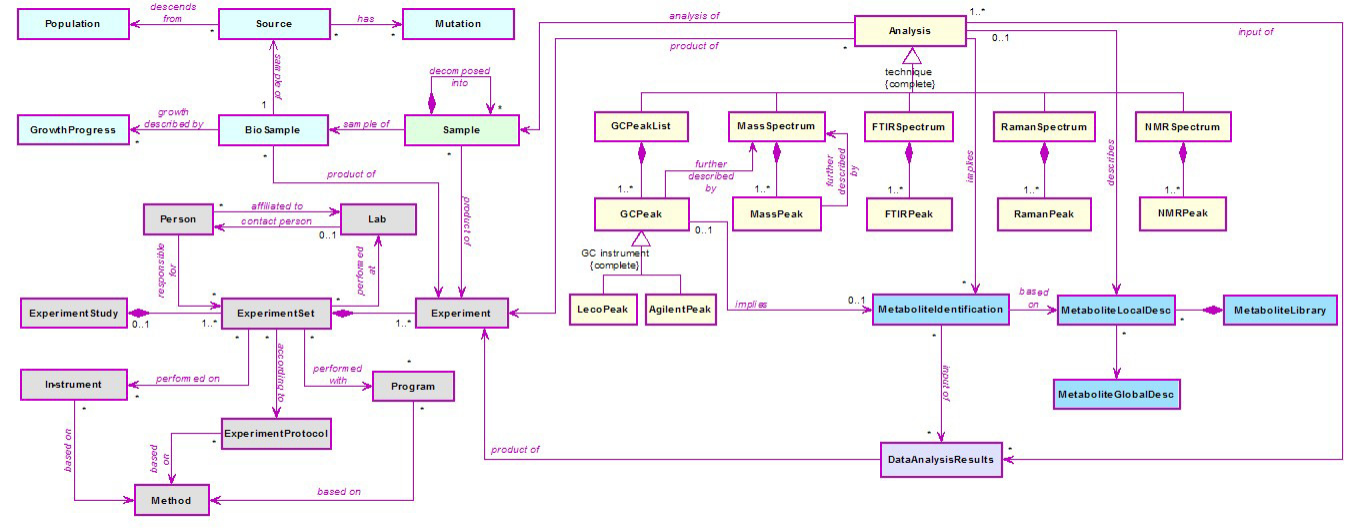
**Detailed overview of MeMo**. The detailed components of MeMo represented by UML classes.

Figure 2 describes a more detailed version of the MeMo model represented by UML classes and their relations.

#### Admin

The central class in the Admin package is ExperimentSet, whose instances are used to represent a homogenous set of scientific experiments. These experiments are performed under the same conditions, which are formally described using the ExperimentProtocol class and each experimental protocol follows a certain Method. Grouping such experiments into sets removes the need to specify these conditions repetitively for each individual experiment. Also, a single Person is in charge of controlling these experiments within a single Lab. Individual experiments, represented by instances of the Experiment class, differ only by their chronological features and in relation to their subjects. Sets of experiments of different types are grouped into an ExperimentalStudy, which summarises a specific metabolomic study. The model does not explicitly differentiate between different types of experiments (i.e. biological sample preparation, chemical sample preparation, analytical experiment and data analysis) at the class level. In other words, a single class is used to describe all four types of experiments, because there are no structural differences at this level. The differences arise only in relation to other classes. For example, an analytical experiment is performed on an analytical Instrument, while data analysis is performed using a Program. Since the same instrument is used to perform a set of analytical experiments, there is a link between the ExperimentSet and Instrument classes. This is in contrast to the link between a single Experiment and the biological sample (the BioSample class in the Biological sample package) it produces.

#### Biological sample

Each metabolomic study concentrates on a specific biological source. An instance of the Source class represents an individual organism (e.g. a specific patient in the case of clinical studies) or a genetically defined strain of a particular organism (e.g. a specific gene-knockout strain of *S. cerevisiae*). In this model, we focus only on the latter case, where each Source instance is linked to instances of the Mutation class denoting the genes that were knocked out/in from a given biological source. Large-scale genomics studies investigate whole populations of related biological sources rather than a single source. For example, there is sometimes a mutant for each gene in functional genomics studies using a model organism such as *S. cerevisiae*. Instances of the Population class are used to describe a studied group of related biological sources. Each Source is used to produce a biological sample (e.g. metabolomic footprint of a given *S. cerevisiae *strain [30]), which is represented using the BioSample class.

#### Chemical sample

A biological sample (BioSample) is prepared as a Sample to be analysed using an analytical Instrument. Such sample is produced by a chemical sample preparation Experiment, which in turn follows a specific ExperimentProtocol.

#### Instrumental analysis

An instance of the Analysis class refers to the results of analyzing a Sample using an analytical Instrument. URL references are used to specify the location of the raw data files. Different classes are used to model different types of data depending on an analytical technique used, e.g. GC, MS, FT-IR, Raman and NMR spectroscopy. Depending on the analytical method, different instruments produce different kinds of spectra (e.g. GCPeakList, MassSpectrum, FTIRSpectrum, RamanSpectrum, NMRSpectrum). Each spectrum is modelled as a list of peaks. Different types of peaks are used for different analytical techniques (e.g. GCPeak, MassPeak, FTIRPeak, RamanPeak, NMRPeak) and they differ in the names and types of *x *and *y *values recorded. No generalisation [e.g. where peak tables consist of generalised *x *(generalising wave number, *m/z*, etc.) and *y *(generalising absorbance, intensity, etc.) data] has been attempted here in order to prevent the loss of specific semantics allowing more detailed representations of specific techniques and the ways in which they can be combined (e.g. for hyphenated techniques such as GC-MS). Different types of peaks will also have different types of additional information attached depending on the analytical Instrument used. For example, GC peaks detected by a GC-MS instrument produced by Leco or Agilent will have different features and these differences are modelled by the corresponding subclasses LecoPeak and AgilentPeak of the GCPeak class. In this manner, the model can be extended easily to support new instruments. Similarly, new types of spectra can be supported by adding new subclasses to the Analysis class as recommended in [31].

#### Data processing

The results of an Analysis can be used to identify different metabolites and their concentrations within the Sample analysed. For example, definitive identification of metabolites detected by a GC-MS instrument is performed by matching chromatographic peaks. This can be performed either using external mass spectra libraries or internal libraries obtained by analysing samples of pure chemical standards. In the latter case, the relevant details are recorded and stored as usual: the samples are described using the ChemicalSample component, the experimental protocols and instrument settings are stored using the Admin component, and the analytical results (e.g. the actual peaks produced) are stored using the appropriate classes (depending on the method/instrument used) in the InstrumentalAnalysis component. In addition, each Analysis produced as a characterisation of a given metabolite is used to create a new item in a MetaboliteLibrary. This item corresponds to an instance of MetaboliteLocalDesc, i.e. a local description of a given metabolite. Each local description of a metabolite is further mapped to its global description (class MetaboliteGlobalDesc), where the general information about the metabolite is stored: its identifiers in other resources, its names, molecular formula and weight, SMILES strings, etc.

When performing metabolite identification, using either an external or an internal library, different parameters quantifying the quality of a match are used depending on a specific instrument and software. These attributes are, therefore, recorded in the instrument-specific classes. For example, when a Leco instrument is used for GC-MS analysis, spectrum similarity and reverse matches are used as such attributes in the LecoPeak class. The actual metabolites identified are modelled by the Metaboliteldentification class, which links the analytical results directly to a local description of a metabolite and indirectly to its global description.

#### Data analysis

Finally, both quantitative (modelled by Analysis and its subclasses) and qualitative (modelled by Metaboliteldentification) data can be further analysed to produce DataAnalysisResults. Such an analysis is performed as an Experiment using some Program, similarly to an analytical Experiment being performed using an Instrument.

## Utility

### Technologies

Once the schema has been developed, it needs to be implemented as a DB. The most commonly used DBs are relational, OO and XML. A UML model can be translated straightforwardly into an OO DB. For example, such an approach has been taken in the development of the GIMS DB used to store genomic and functional data [27,29]. It can also be relatively easily translated into a relational or XML DB. For instance, the OO model for functional genomics described in [28] has been implemented as an RDB. The PEDRo model for proteomics [18] is used to convert data into the corresponding XML format, and the XML files so produced can be stored in a DB of the user's choice. The ArMet model for plant metabolomics exists in parallel as both an XML version and an RDB [22].

OO DBs have never really gained widespread acceptance. On the other hand, XML [32] as an exchange format has become a predominant means of information modelling in biosciences [33], allowing the design of customised markup languages, web-enabled data exchange and full data management including modelling (schemas defined by DTD or XML Schema), storing (XML documents which can be stored as files or in DBs) and querying (XML query languages, e.g. XQuery, XPath).

XML is particularly suitable for structurally complex data that cannot be easily represented by tabular structures. In an RDB, an additional table is usually created to support equivalents of nested XML elements in order to represent variable hierarchical structures. This could significantly increase the number of tables required, thus negatively affecting the transparency of the DB and consequently its maintenance and performance. The most natural way to store XML data is in an XML-native DB, which can store and index XML documents directly (unlike XML-enabled DBs, which require their mapping to the underlying relational or OO format). However, XML DBs currently cannot deliver the necessary speed of access [34], which is critical for many bioinformatics tasks involving huge volumes of data, as is the case in large-scale metabolomic studies. On the other hand, RDBs represent a mature technology when it comes to speed of access. Indeed, the vast majority of biological DBs are implemented using the classic RDB management systems [5,35], e.g. PostgreSQL, MySQL, Oracle, DB2, SQL Server, etc., and this fact needs to be taken into account when interoperability with the existing DBs needs to be supported. Other features used to determine a specific choice of a DB type include flexibility in terms of generality, extensibility, ease of access and portability.

The current state of computer technology does not offer a perfect solution. One needs to weigh carefully the pros and cons of each technology in the context of typical uses of the given model. Thus, the MeMo model has been implemented using two formalisms, XML and SQL, which can be used in parallel for different tasks, but most importantly have also been combined in a hybrid approach that has enabled us to unify their benefits.

### Implementation

The XML version of the MeMo model is an XML schema [36] written in the XML Schema language. It defines the structure of XML documents that can be used to store the metabolomic data in a file or XML DB, or to support data exchange in the XML format (e.g. similar to [37]). The schema has been developed using a modular approach and taking full advantage of XML namespaces to resolve the problems of ambiguity and name collision. The overall XML schema follows the global structure of the MeMo model shown in Figure 1, i.e. there is a separate schema for each component. The administrative schema further includes the schemas describing the structure of experimental procedures as well as instrument settings for each of the supported analytical techniques (MS, GC, FT-IR, Raman and NMR spectrometry, etc.). The modularity of the schema makes it more manageable, since it enables independent development of different modules and the possibility of their independent usage (e.g. on their own or as part of other schemas). Currently, the XML schema is used to improve the usability of the implemented RDB (explained later). Further work is underway to configure the Pedro tool [38] to capture the metabolomic metadata in the XML format according to the given schema. As Pedro is XML-schema driven, its flexibility allows instant changes in the schema to be reflected in the data capture forms.

**Figure 3:**
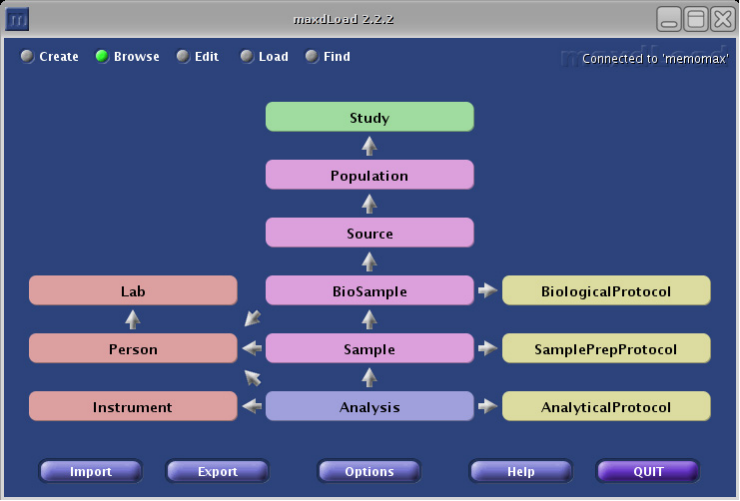
**Screenshot of the MeMo schema in maxdLoad2**. The maxdLoad2 interface hides the complexity of the schema and some implementation details from the user in order to allow user-friendly data capture and dissemination of metabolomic data.

In parallel to the XML schema, we implemented MeMo as an SQL schema and thence as an RDB using PostgreSQL. Many biological DBs contain a large number of tables causing difficulties in locating information [39]. One of the principles which can be used to reduce the number of tables is generalisation, where a single table is used to represent similar entities leading to a smaller and more flexible schema. For example, we use the same set of tables to structure administrative information related to all four types of experiments: biological and chemical sample preparation, instrumental and *in silico *analysis. In order to support a user-friendly interface to the RDB, the maxdLoad2/maxdBrowse suite [40] is being configured to reflect a user-orientated representation of the MeMo model (Figure 3).

Nelson et al. [39] recommend leaving a subset of data in a flat file format as opposed to parsing them into the tabular structures. This decision should be reviewed with respect to the ways in which the data will be typically used. For example, if the data are primarily accessed in their entirety, then parsing them into the DB may be unnecessary. We use a similar approach in the MeMo DB, namely, we use an RDB with XML extensions where appropriate. Tables are used to represent fixed structures, while XML is used to model highly variable structures. Instead of using flat files we store XML documents in the table fields. Most entities are represented directly by tables. Other entities are represented by textual fields further structured by XML. The criteria used to choose between the two representation approaches include structural stability, hard vs. soft coding, querying vs. browsing, access speed and ease of maintenance.

#### Tabular format

In a well-designed RDB, each row in a table should represent a single instance of the entity type modelled by the table. In the case of metabolomics, when modelling the analytical spectra of various types one is faced with a decision of viewing a **peak **as an *entity *on its own or simply as an *attribute *of a spectrum. In the latter case, a spectrum would be represented by the following structure described in SQL (all data types provided in the SQL definitions are used for illustrative purposes only; different types of spectra may require different data types):

CREATE TABLE spectrum

(

**Figure 4:**
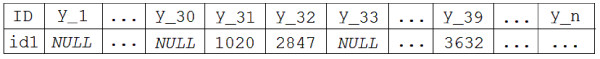
**Horizontal spectrum representation**. Each row represents a spectrum, where "*y*-columns" are used to store the *y*-values for the corresponding "*x*-coordinates".

ID VARCHAR(50) NOT NULL,

y_1 INT,

y_2 INT,

...

y_n INT,

PRIMARY KEY(ID)

);

Each row represents a spectrum, where "*y*-columns" are used to store the *y*-values for the corresponding "*x*-coordinates" (see Figure 4). Although this is a "natural" representation for human users, who are used to seeing spectra displayed in such a format, it does not represent good design practice when it comes to the RDBs. First, the *x*-values are implicitly represented by the column names and not by their own values. Such an approach would be acceptable if, across different spectra: (1) the increment of the *x*-values was constant, and (2) the range of *x*-values was constant. In that case the total number of the *x*-values would be fixed as well. However, in order for such a table to be easily manipulated the total number of the *x*-values should also be relatively small. Still, the user would not be able to take the advantage of the aggregate functions (e.g. summation – SUM(), finding minimal – MIN(), average – AVG() or maximal – MAX() value in SQL to process a spectrum (e.g. in the case of mass spectrometry determining the total ion current by summing all intensity (*y*) values), because these functions operate over multiple rows and not columns as required.

Since a peak itself has internal structure (*x *and *y *values, e.g. *m/z *and *intensity *in the case of mass spectrometry), we are in favour of a representation in which a peak is treated as an *entity *and represented by a separate table (peak) different from the one used to represent a spectrum. This is formally described by the following definitions in SQL:

CREATE TABLE spectrum

(

ID VARCHAR(50) NOT NULL,

x_min INT,

x_max INT,

PRIMARY KEY(ID)

);

CREATE TABLE peak

(

spectrum_ID VARCHAR(50) NOT NULL,

x INT NOT NULL,

y INT NOT NULL,

PRIMARY KEY(spectrum_ID, x),

FOREIGN KEY(spectrum_ID) REFERENCES spectrum(ID)

);

Given an *x*-value, a single peak is efficiently retrieved by the following query:

SELECT *

FROM peak

WHERE spectrum_ID = 'given spectrum'

AND x = x_value;

A whole spectrum of *x*- and *y*-values is retrieved via a simple SQL query:

SELECT *

FROM peak

WHERE spectrum_ID = 'given spectrum';

However, the high-dimensional nature of analytical data can cause efficiency problems. For example, mass spectra produced by a method such as DIMS [30] tend to produce a vast array of data, e.g. a *y*-value for all integer *x*-values between 45 and 999. Some instruments that give so-called "accurate mass" measurements have many more *m/z *values. This can pose considerable memory requirements to handle the retrieved data and consequently prolong the retrieval time. For example, the first query took only ≈30 ms compared to ≈140,000 ms consumed by the second query. (All times reported have been obtained for DIMS data, *m/z *ranging from 45 to 999, stored in a PostgreSQL DB. The exact times will vary on different systems. They are provided only to illustrate the relative differences in executing the given queries.) The retrieval time is additionally extended (≈147,000 ms in total) when it is required for the *x*-values to be ordered:

SELECT *

FROM peak

WHERE spectrum_ID = 'given spectrum'

ORDER BY x;

When a large number of spectra need to be processed, they need to be retrieved much faster. We adopted a caching approach, where some of the features are extracted in advance and stored for future use providing a quick snapshot of a spectrum. For example, we extend the definition of the spectrum table as follows:

CREATE TABLE spectrum

(

ID VARCHAR(50) NOT NULL,

x_min INT,

x_max INT,

avg_peak FLOAT,

max_peak INT,

total_peaks INT,

PRIMARY KEY(ID)

);

Additional attributes avg_peak, max_peak, total_peaks store the values for the average and maximal *y*-value and the total number of peaks recorded. Other attributes may be stored as well depending on the nature of the analytical technique used (e.g. total ion current for mass spectrometry). These attributes are redundant in the sense that they can be calculated from the peaks table, e.g. total_peaks can be calculated as:

SELECT COUNT (*)

FROM peaks

WHERE spectrum_ID = 'given spectrum';

However, these values are often accessed and it makes sense to store them in advance and simply retrieve them when necessary:

SELECT total_peaks

FROM spectrum

WHERE spectrum_ID = 'given spectrum';

since the latter query consumes considerably less time (≈30 ms) compared to the former one (≈141,000 ms).

#### Compact ASCII format

When a whole spectrum needs to be retrieved and processed, it is useful to treat it as a bundle of data as opposed to a collection of data items (i.e. peaks). For this purpose, we use a compact (or compressed) spectrum representation, which is stored in the spectrum table as a single attribute (peaks). The following assumptions apply to an individual spectrum: (1) the range of *x*-values is known, and (2) all *x*-values are recorded with the same precision *p *(the number of digits to be recorded after the decimal point). The compact format takes advantage of the fact that all *x*-values can be generated from the initial value using 10^-*p *^as an increment: *x*_*n *_= *x*_0 _+ *n*·10^-*p*^, and therefore need not be explicitly stored. Instead, only the initial value and the increment are stored, while the *y*-values are stored in correspondence to the ascending order of *x*-values. In addition, when a spectrum is sparse (e.g. mass spectra obtained through GC-MS), instead of storing the *NULL *values explicitly, they can simply be skipped by recording the number of consecutive *NULL *values. We define a compact spectrum format by the following regular expression:

(<separator> <value>)^+ ^<separator>

where <value> is a numerical value and <separator> stands for any of the following characters: |, #, + and -. The separators indicate how the *y*-values can be constructed from the given numerical values. The vertical bar (|) is used to separate the actual *y*-value from the previously stored value. For *n *consecutive *NULL *values, the hash sign (#) is used as a separator followed by *n*. The plus (+) and minus (-) signs are used when the difference between the *y*-value to be stored and the last stored *y*-value is shorter (in the number of digits) than the actual *y*-value to be stored. In such case, + or - are followed by the absolute value of the difference (so that additional space can be saved) and they indicate that the current *y*-value can be obtained by adding or subtracting the current value from the last recorded *y*-value. When decimal numbers are used, they should be multiplied by 10^*p *^(where *p *is the number of digits recorded after the decimal point) in order to remove the decimal point and, more importantly, the leading zeros, thus reducing the number of digits to be stored. Additional space can be saved by recording the numbers using a bigger base (e.g. 16 to record *x*- and *y*-values as hexadecimal numbers), since fewer characters (i.e. bytes) are needed to represent individual numbers (the programs for packing and unpacking the compact spectrum representation are available at the MeMo web site).

**Figure 5:**
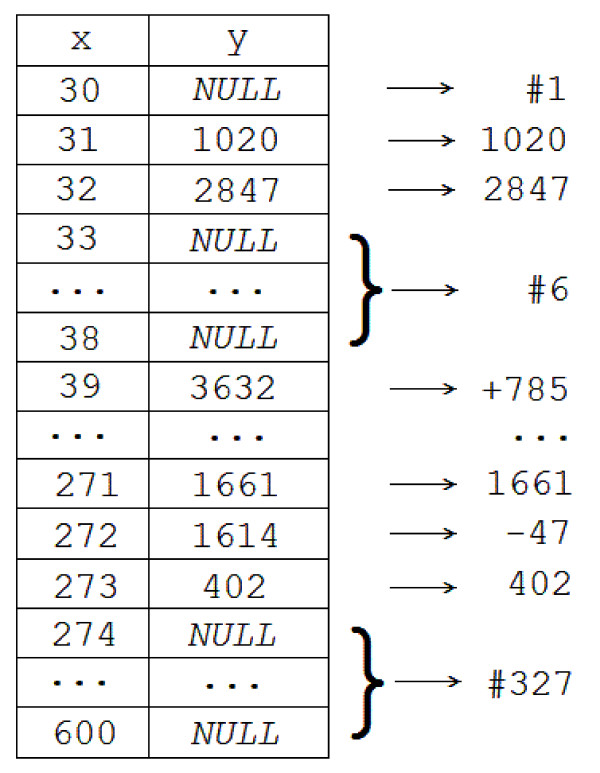
**Compact spectrum representation**. An example of compacting a mass spectrum.

We use an example to illustrate the compact format. Given a mass spectrum (see Figure 5), where *m/z *values are integers (which implies that the increment to be used is 1) ranging from 30 to 600, the compact representation is as follows:

#l|1020|2847#6+785 ... |1661-47|402#327|

A more compact representation is obtained using 36 as a base:

#1|*SC*|273#6+LT ... |1*A*5-1*B*|*B*6#93|

The compact spectrum representation is stored as a text field (peaks) in the spectrum table from which it is retrieved in ≈30 ms as follows:

SELECT peaks

FROM spectrum

WHERE spectrum_ID = 'given spectrum';

We mentioned earlier that the same time is required to retrieve a single peak from the peak table. However, accessing a specific peak in the compact spectrum representation requires additional time for the compact representation to be parsed. We therefore see justification for storing the spectra in both formats and using one or the other depending on a specific application. For example, if a spectrum needs to be presented graphically, then the compact representation is more suitable, since the peaks are already ordered by the *x*-values and the spectrum would be sequentially processed peak by peak so no random access to peaks is actually required.

Let us contrast our compact spectrum representation with the binary representation, which is similarly used to save space when storing spectra. For example, the mzData [41] format supports storing mass spectra in the binary format. The *x*- and *y*-values are stored separately as byte arrays for the recorded peaks. Depending on the precision (single or double), 4 or 8 bytes are used for each *x *or *y*-value, which is 8, 12 or 16 bytes per peak. In this format, the number of bytes used per peak is constant, while in our format it varies depending on the number of digits (each represented as an ASCII character) in the *y*-value of the peak. Therefore, none of the formats generally outperforms the other in terms of the space requirements. In general, it is expected that the binary format will do better when the *y*-values are represented as floating point numbers with high precision. We compared the space requirements of the two formats using the mass spectra from two studies using GC-MS and DIMS, respectively. Mass spectra obtained through GC-MS tend to be sparse as opposed to mass spectra obtained through DIMS. For 223,601 mass spectra (*m/z *ranging from 30 to 600) obtained through GC-MS, the binary format and our compact representation required 743 and 450 bytes on average, which means that our format saved 39% of the binary format. When 36 is used as a base for the compact representation, the average number of bytes used goes down to 362, which saves 51% of the binary format. For 762 mass spectra (*m/z *ranging from 45 to 999) obtained using DIMS, the binary format and our compact representation required 7640 and 3154 bytes on average, which means that our format saved 59% of the binary format. When 36 is used as a base for the compact representation, the average number of bytes used goes down to 2537, which saves 67% of the binary format. Judging by these data, our format works better for some types of the mass spectrometry data, though this is not the case in general. Therefore, both formats should be used sensibly, depending on the nature of the data and specific applications.

#### Embedded XML format

As explained earlier, a spectrum is a series (usually several hundreds) of data points ordered by the *x*-values, each mapped to the *y*-value registered, for which a tabular structure (e.g. a table with separate columns for *x *and *y*) is a natural choice of format. The structure of this type of data is thus fixed and can be hard-coded into the DB using the tabular format. Further, in a data-mining scenario, the peaks need to be accessed many times in order to make not only comparisons within a single spectrum but also across spectra acquired for different samples analysed. RDBs excel at accessing well-defined tabular structures containing multiple elements with identical features [5]. In the metabolomic studies, the spectral data are actively queried rather than simply being browsed, and hence fast access is not only desirable but is in fact necessary for real-time applications. We conclude that the tabular format is suitable for the spectral type of data.

**Figure 6:**
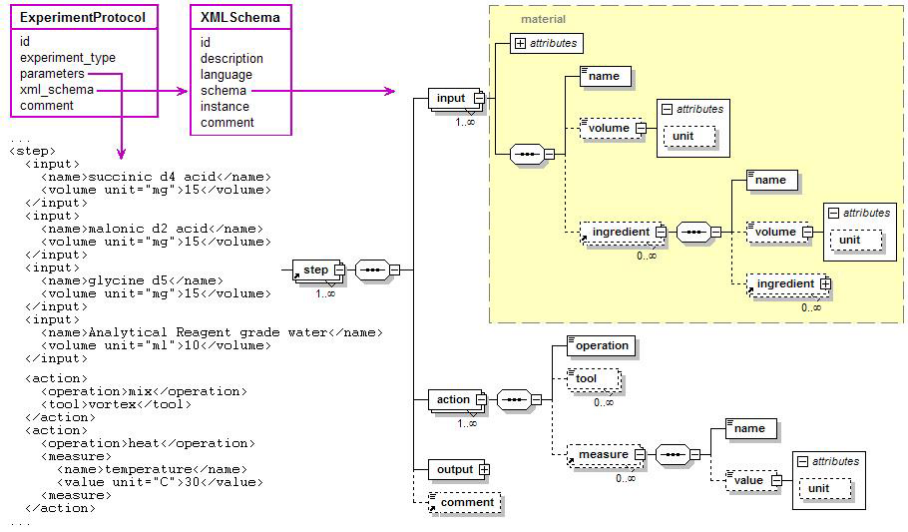
**An embedded XML document**. Embedding an XML document representing an experimental protocol into the MeMo database. The XMLSchema entity is not included in the model (Figures 1 and 2), because it is a part of the implementation. The given schema is depicted using XMLSpy 2005 .

We now consider the experimental protocols as a type of data with a significantly different nature. First, there are four types of experiments whose protocols are defined in a different manner. For example, biological and chemical sample preparation experiments typically consist of certain steps, each of which corresponds to some actions performed on input material in order to produce an output (Figure 6). On the other hand, experimental protocols for analytical experiments refer to particular instrument settings, which vary significantly across different analytical techniques and instruments. Similarly, experimental protocols for data-mining experiments describe the parameter settings for the particular data mining approach applied.

In the intended metabolomic studies, such types of data would be used for documentation and teaching purposes in order to allow the experiments to be reproduced and their results compared. In such cases, the given data are browsed as a single object rather than being queried parameter by parameter [31]. It is possible to use a single table (e.g. with two fields for each parameter referring to its name and value) to encode such information. However, different parameters may have values of different types. It is also difficult to restrict the values applicable to specific parameters when a single field is used as a generic holder for all parameters. Consequently, various types of internal inconsistencies may arise, which otherwise could be prevented by enforcing the data integrity automatically through typing, referencing, normalisation, etc.

Further, some parameters may have specific attributes (e.g. units) and an approach in which a separate column is used for each of them, would lead to a high number of *NULL *values. It is also difficult to maintain the groups of related parameters in tabular representations: a separate table for each group of highly variable data would lead to an explosion of tables. For example, GC-MS parameters can be divided into 5 groups (gas chromatograph, mass spectrometer, autosampler, electronics operating, data processing), each containing 12 parameters on average [42], which can further be structured resulting in a hierarchy of 5 levels. A fully structured representation of the current GC-MS instrument model would require around 25 tables. When this number is multiplied by the number of different analytical techniques, it demonstrates how the complexity of the DB can easily scale out of control by adding new features, some of which cannot be predicted during the DB design. This could obscure the focus of a DB for metabolomics, making it unmanageable by both the administrator and the user.

On the other hand, hierarchically organised data can be stored naturally and conveniently in XML. Moreover, the XML Schema language supports a referencing mechanism [43] analogous to the foreign keys used in RDBs, which can be used to support even more complex structures. Further, XML Schema enables strong typing by supporting not only the standard data types, but also sophisticated constraining mechanisms such as a range of values, enumeration, or even a regular expression describing the allowable patterns.

Once defined, XML schemas can be used to validate the content of the corresponding XML documents. This is a huge advantage for storing some types of data as a single structured entity as opposed to using a flat file. Other general advantages of embedding XML documents to represent variably structured entities into the RDB include easy manipulation (i.e. storing and retrieval) of such entities, readability by both users and XML-aware computer applications, extensibility, etc. For example, in order to retrieve the instrument settings used in a specific experimental protocol, one would simply need to select the corresponding field from the given table:

SELECT parameters

FROM ExperimentProtocol

WHERE ID = 'given protocol';

without the need to join multiple tables describing different types of instrument settings, which would be cumbersome if different tables were used for different types of instruments (i.e. it would require the user to resort to the DB documentation frequently). Further, the XML information presented to the user is more readable than is a long row of attribute-value combinations due to the self-descriptive property of XML. For example, even a novice user would be able to interpret the following XML content intuitively:

<temperature unit="C">250</temperature>

as a temperature of 250°C. Moreover, the relation between individual elements can be retained by observing their nested organisation. Consider, for example, the XML excerpt shown in Figure 6: step is part of a block, inputs and actions are parts of a step, etc. Even the order among the elements of the same type can be used to infer, for instance, that the mixture of inputs should be first mixed (with a vortex mixer) and then heated (to 30°C). Although reasonably intelligible to humans, the given information is still machine-readable. XML data can be parsed, queried and processed by computer applications. Therefore, metadata stored in the XML format can be automatically processed (parameter by parameter) to account for effects of methodological changes on the analytical data. Of course, the access to XML elements will still be comparably slower than accessing specific fields in a table of an RDB [44], but the metadata would need to be accessed only once per large set of data proceeding from it (e.g. one protocol used in a single study of thousands of gene knockouts).

Further, in order to support an additional instrument type, the corresponding XML schema can be defined independently (thus not causing any rippling effects), stored in the table of XML schemas, the parameters field re-used to store specific instrument settings and linked to the schema according to which the content of the parameters field is structured (Figure 6). Note that the latter is not important for the DB user, who can read the given XML document without the schema. It is given in order to signal to computer applications how to parse the document. Also note that these changes affect only the content of the RDB and not its structure. Neither the user nor the administrator would be affected by these extensions to the DB although its scope would definitely increase.

#### External references

Another important decision made during the implementation was not to store the raw data directly into the DB for reasons similar to those used to suggest that images should not be stored physically into a DB [39]. Although storing the raw data into the DB would prevent their loss during any file system reorganisation, it would significantly increase the size of the DB. In that case, larger servers and a more powerful DB management system would be required in order to handle the sheer amount of data. A more practical solution is to store only a reference to the raw data file, which itself should be stored externally.

### Data integration and interoperability

The scope of any biological DB should be clearly defined and aimed at providing the essential information for a specific problem [5]. It does not mean that a single DB would be sufficient to solve any problem at hand. For example, the MeMo DB will be used to learn to classify individual genes in *S. cerevisiae *based on the corresponding metabolomic data and the associated metadata. The metabolomic data (e.g. mass spectra) on their own do not necessarily provide the information needed to learn the gene functions. Supervised learning algorithms need an annotated set of data together with some knowledge about at least some of the genes and their functions [24]. In addition, knowledge about the metabolites identified from the spectra should help the automatic reasoning process. Obviously, the metabolomic data need to be integrated with the background knowledge sources. Our model includes a background knowledge module intended to be used for this purpose and the DB currently integrates data from several external sources (SGD [45], CYGD [46], KEGG/LIGAND [47] and PubChem Substance [48]).

While it is useful to integrate multiple DBs physically in order to build a comprehensive subject-oriented DB, not all relevant DBs can be integrated in this manner bearing in mind their growing number and frequent changes to their content [7]. Still, the inherently integrative nature of biology [11] requires the content of apparently diverse DBs to be correlated [39,49]. Therefore, various DBs need to be semantically integrated in a more flexible framework. The absolute minimum for practical DB integration is maintaining the cross-reference information (e.g. SGD identifiers, EC and CAS numbers are used for this purpose in MeMo). By contrast, a more sophisticated semantic integration is based on an ontological description of DBs (including both their structure and the content) [50]. For instance, SEMEDA [51] provides semantically integrated access to DBs based on the ontologies and controlled vocabularies, which can be collaboratively edited [35]. The basic principle used in SEMEDA consists of semantic DB definitions, which map DB tables, attributes and attribute values to the concepts in the ontology. These definitions are used to generate cross-references for all attributes that share the same semantics and use the same vocabulary. The generated cross-references can then be used to link DBs automatically, which can be queried through SEMEDA without requiring knowledge of the structure or any technical details about the underlying DBs.

We have used SEMEDA's own custom ontology to annotate the MeMo DB semantically. The actual integration of MeMo into SEMEDA only requires JDBC (a Java application programming interface that provides connectivity to a wide range of SQL DBs [52]) access to be granted to our DB. Meanwhile, a detailed metabolomics ontology is needed to provide formal semantic support for experimental metadata aspects. The MSI-Ontology Working Group [53] has been appointed by the Metabolomics Society [21] in order to facilitate consistent semantic annotation of metabolomics experiments, which will consequently provide a basis for the integration of data across disparate metabolomics databases.

Apart from integrating the MeMo DB with other relevant data resources, there is a need to make it compatible with relevant data formats so that the data can be exported from the DB and shared in fairly standardised formats, which can be interpreted not only by human users but, more importantly, the software tools that support such formats. We currently support an export option to the mzData format [41] (the source code is available at the MeMo web site). This format focuses on the data exchange issues in the field of mass spectrometry, a technique directly related to metabolomics. We plan to support other emerging standards related to metabolomics, either partially (i.e. covering certain aspects of metabolomics, e.g. mzXML [54], which also relates to mass spectrometry or CCPN for NMR [55]) or fully (e.g. any format emerging from the SMRS group [20] and the Metabolomics Society [21]).

## Discussion

We have described MeMo, a metabolomics model and data format for yeast, which is MIAMET-compliant [19], i.e. supports the description of the minimum information that should be reported about metabolomic experiments including biological and experimental metadata, a variety of analytical techniques and metabolite specifications. An effort will be made to make MeMo compliant with the emerging standards [20,21]. As a model, it is closest to ArMet [22], which offers an analogous model for plants. The two models have been developed in parallel with occasional collaboration. Thus, MeMo conforms to the standard proposed in ArMet, which is seen as a formal means of metabolomic experiment tracking and management. Although ArMet is currently being used as one of a number of inputs to the MSI, its utility has originally only been demonstrated for plant-based experiments. MeMo shows how a new organism can be incorporated into ArMet's basic framework. In addition, ArMet puts no emphasis on any specific implementation. On the other hand, strong emphasis is given to the actual implementation of MeMo (using SQL and XML) with a specific goal of associating metabolomic data with *S. cerevisiae *genes in order to provide insight into their functions through computational analysis. While data modelling represents theoretical research, an implementation of a data model puts a specific model into practice.

During the development of the MeMo DB, we followed the best practices recommended for the design of biological DBs [39]. A distinct characteristic of the MeMo DB is the way in which the problem of scope creep is tackled: structurally complex and variable entities (e.g. experimental protocols) are soft-coded into the DB through the use of XML in order to represent them as a single DB object (table column) whose structure can be formally interpreted via an extensible set of XML schemas. This approach both greatly reduces the complexity of the RDB and facilitates its scalability.

Semantic annotation of the MeMo DB facilitates its integration with other related biological DBs as the first step towards realizing the goals of SB. The next issue would be to support the integration at the model level as well. In general, the existing omics models (e.g. [15,18,44] as well as MeMo) share a crude structure that enables the storage of both experimental data and associated metadata. There is thus room for standardising common parts across different omics models and re-using them in a single omics model (e.g. SysBio-OM [56]). FuGE-OM [57] is under construction to enable common data standards for all levels of functional genomics to be developed [28].

## Conclusion

The MeMo schema is fully operational and has been in use to support the pressing need to store, manage and disseminate large amounts of curated metabolomic data efficiently. However, it needs to support the ongoing standardisation efforts as well as to make room for the incoming methods, instruments and data resources. Therefore, it is not only desirable, but absolutely necessary for MeMo to evolve together with the domain it describes. The key traits of MeMo, its modularity and extensibility, make it highly adaptable to the inherent fluidity of metabolomics and related omics domains.

## Availability and requirements

Project name: MeMo

Project home page: 

Operating system(s): Platform independent

Programming language: SQL

Other requirements: Access to SQL database

License: The SQL schema is freely available

Any restrictions to use by non-academics: None

## Abbreviations

ArMet architecture for metabolomics

DB database

DIMS direct injection mass spectrometry

DTD document type definition

FT-IR Fourier transform infrared

FuGE-OM functional genomics object model

GC gas chromatography

GC-MS gas chromatography – mass spectrometry

GIMS genome information management system

JDBC Java database connectivity

MBDC molecular biology database collection

MeMo metabolomics model

MIAME minimum information about a microarray experiment

MIAMET minimum information about a metabolomics experiment

MIAPE minimum information to describe a proteomics experiment

MS mass spectrometry

MSI metabolomics standards initiative

NMR nuclear magnetic resonance

OO object-oriented

RDB relational database

SB systems biology

SEMEDA semantic meta database

SMRS standard metabolic reporting structure

SQL structured query language

UML unified modelling language

XML extended markup language

## Authors' contributions

The analysis was performed jointly by IS, WD, HJ, NH, SGO and DBK. IS implemented the model and the database. GV and AT implemented a draft version of the database interface. IS drafted the manuscript and revisions were made by all co-authors.
